# Mandibular versus Maxillary Desmoplastic Fibroma: A Pooled Analysis of World Literature and Report of a New Case

**DOI:** 10.1055/s-0044-1801277

**Published:** 2025-03-12

**Authors:** Taiseer Hussain Hassan Al-Khateeb

**Affiliations:** 1 Department of Oral and Maxillofacial Surgery, Jordan University of Science and Technology, Irbid, Jordan

**Keywords:** fibromatosis, aggressive, desmoid, mandible, maxilla, jawbones

## Abstract

The purpose of this study is to delineate differences between mandibular and maxillary desmoplastic fibroma (DF) via analysis of published cases. Details of cases were analyzed for distribution, demographics, presentation, treatment, and follow-up. Between 1961 and 2022, 195 cases were reported, averaged 2.7 annually. There were 159 (81.5%) mandibular and 36 (18.5%) maxillary cases. The posterior mandible was most commonly affected (91.2%; *p* < 0.05). Maxillary DF mostly involved the anterior region (53.1%; *p* < 0.05). The female:male ratio was 1:1.3 (1:2 mandibular and 1:4 maxillary), and the average age was 13.5 years (12.1 mandibular and 20.5 maxillary) with a peak frequency in both jaws in the first decade of life ( *p* < 0.05). Mandibular cases mostly affected whites, and maxillary cases affected a higher percentage of Asians ( *p* < 0.05). Maxillary cases caused more pain and intraoral ulceration ( *p* < 0.05). The combined cure rate of all treatment modalities for mandibular and maxillary cases was 74.8 and 81.5%, respectively. The most effective single-modality treatment for DF of both jaws was surgery ( *p* < 0.05). To the best of our knowledge, this review is the first to delineate difference between mandibular and maxillary DF. This work will aid attaining an improved management protocol of this uncommon disease.

## Introduction

Desmoplastic fibroma (DF) is a rare monoclonal, fibroblastic proliferation disease characterized by an inconstant and frequently unpredictable clinical course. It was first reported by Jaffe in 1958 1 ; he described multiple sites of involvement in the tibia, scapula, and femur. DF is classified as [D48.1] in the International Classification of Diseases. According to the World Health Organization, DF is a “clonal fibroblastic proliferation that arises in the deep soft tissues and is characterized by infiltrative growth and a tendency toward local recurrence but an inability to metastasize.” 2 It occurs at intra-abdominal and extra-abdominal locations, and constitutes a soft tissue mass arising at any part of the body in different types of connective tissues including muscle, fascia, and aponeurosis. DF is a noticeably uncommon disease with an incidence 5 to 6 cases per 1 million of the population per annum, 3 with a peak age of 30 to 40 years mainly in women at reproductive age. 4 DF can lead to severe pain, functional impairment, and, rarely, can be a life-threatening condition. Spontaneous regression, long-lasting stable disease, and disease progression can occur, but reliable predictive features are unfortunately lacking. Histologically, DFs are composed of monoclonal spindle-shaped cells separated by an abundant collagenous matrix. At a genetic level, DF comprises at least two different clinico-pathological entities: sporadic DF and DF associated with germline mutation of *APC* . 4 The association with the *APC* gene mutation can result in multifocal DF, occasionally leading to mortality.

DF of bone is extremely rare, it presents as a locally aggressive bone tumor composed of bland spindle cells in an abundant collagenous stroma, with histology indicative of desmoid-type fibromatosis. 5 There are reports of desmoid fibroma of bone presenting a *CTNNB1* mutation: one p.Thr41Ala and the other one is p. Ser45Phe alteration, with both mutations exhibiting nuclear β-catenin immunostaining. 6 The earliest reported case of jawbone involvement by DF was by Connolly 7 in 1961. Afterwards, other cases of DF of jawbones have been reported either as sporadic case reports or as case series. Despite the numerous case reports, there is a definite paucity of reviews that thoroughly analyze DF of jawbones particularly when delineating difference in behavior and treatment outcomes of this tumor between mandibular and maxillary bones. The goal of this study was to further characterize DF of jawbones and illustrate the clinical features that differentiate mandibular from maxillary DF. This article offers a comprehensive literature review of published cases of DF of jawbones during the past 61 years (between 1961 and 2022), with the addition of one mandibular case.

## Case Report

The father of the boy (FO) signed an informed consent to publish his son's case without identifiable face details.

An Arab Jordanian boy (QFO), aged 1 year and 9 months, was referred from the Pediatrics Department to the Maxillofacial Unit at King Abdulla University Hospital of Jordan University of Science and Technology, Irbid, Jordan. He presented with a swelling in the right submandibular area of 20-day duration. The boy was the first part of a twin and a product of cesarean delivery after uneventful pregnancy. He was otherwise healthy with no history of other lumps, infections, trauma, fever, malaise, or weight loss.

Initial physical evaluation showed a 3 × 4 cm, firm, and nontender mass involving the right submandibular area, attached to angle of mandible. The overlying skin was normally intact with an ordinary appearance. The ipsilateral and contralateral submandibular lymph nodes were small, nontender, and movable (in line with standard findings in children). Other ipsilateral and contralateral cervical lymph nodes were not palpable. General physical examination was within normal limits, with no signs of hepatomegaly, splenomegaly, or axillary or inguinal lymph node enlargement. Routine laboratory tests (complete and differential blood counts, liver and kidney function tests) revealed no abnormality.

**Fig. 1:**
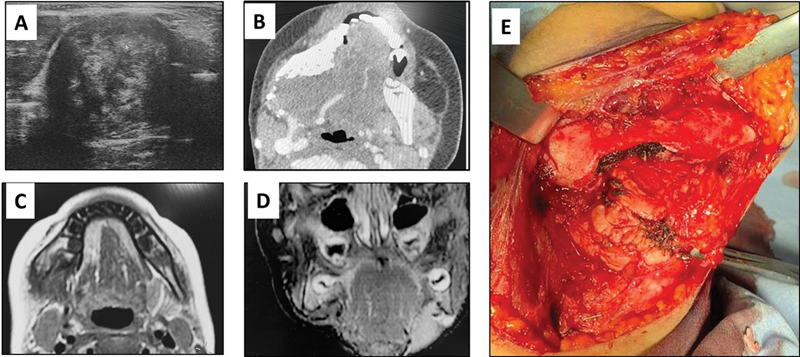
The case reported. ( **A** ) Ultrasound scan at initial presentation showing a well-defined heterogonous soft tissue mass; ( **B** ) CT scan at initial presentation showing heterogonous soft tissue mass lesion associated with erosion and destruction of angle and body of mandible; ( **C** ) follow-up MRI scan showing tumor shrinkage in size; ( **D** ) MRI scan after last dose of chemotherapy showing great shrinkage in size; ( **E** ) total excision of the tumor along with marginal resection of the lower border of the mandible. CT, computed tomography; MRI, magnetic resonance imaging.

Ultrasound (US) of the lump showed a well-defined heterogonous soft tissue mass invading the lower border of the mandible with a size of 3.8 × 3.1 cm ( Fig. 1A ). Computed tomography (CT) of the neck, chest, and abdomen-pelvis (NCAP protocol) was done and showed a 3.3 × 3.9 × 4.1 heterogonous soft tissue mass lesion at the right masticator and submandibular spaces associated with erosion and destruction of ipsilateral angle and body of mandible with interrupted periosteal reaction and small bony fragments ( Fig. 1B ), raising the possibility of rhabdomyosarcoma versus bone sarcoma. True-cut biopsies from the mass were taken, and came back as a benign myofibroma with aggressive features. Incisional biopsy, done under general anesthesia, showed long twirling bundles of fibers, with abundant bland spindle cells, and focal areas of foamy macrophages and areas of hemorrhage. Immunohistochemistry studies showed positive cellular expression for SMA, desmin, and β-catenin, but was negative for S-100, CK, C-kit, MUC 4, CD34, and calretinin. The final diagnosis was “aggressive desmoid-type fibromatosis.”

The case was discussed at head and neck oncology board and the decision was to treat with neoadjuvant chemotherapy to shrink the lesion size, followed by surgical removal of any remaining lesion. The patient was started on bi-weekly chemotherapy, via Port-A-Cath, and the protocol consisted of vinblastine 6 mg/m ^2^ and methotrexate 30 mg/m ^2^ . 8 Over a period of 6 months, 53 cycles were given regularly except for one period of interruption (after dose 30) as parents refused to attend the hospital because of coronavirus disease 2019 pandemic. Tumor shrinkage in size was evident on follow-up magnetic resonance imaging (MRI) scans ( Fig. 1C ). One month after the last dose of chemotherapy, MRI scans showed that the lesion has greatly shrunken in size to 1.5 × 0.7 cm ( Fig. 1D ). Five months after the last dose of chemotherapy, total excision of the tumor along with marginal resection of the lower border of the mandible extending from the right parasymphysis to the right angle was performed, maintaining the continuity of the mandible ( Fig. 1E ). At the time of writing this manuscript (2.5 years postoperatively), the patient remained free of tumor.

## Methods

An English language search of the three databases with available access at our institution (National Library of Medicine [PubMed], Scopus, and EBSCO Discovery Service [EDS] search), since 1961, was conducted looking for cases of DF of jawbones. All literature searches were performed during November 2022. For every database searched, all fields were searched for any of the search terms: *aggressive fibromatosis, desmoid fibromatosis, desmoplastic fibroma, desmoid-type fibromatosis, desmoplastic fibromatosis, fibromatosis, infantile fibromatosis,* and *juvenile fibromatosis* AND one of the following terms: *gnathic, jaw, jawbone, mandible,* and *maxilla.* Repetitive citations for the same article were noticed and repetitions were excluded. Multifocal DF is genetically different from sporadic DF (multifocal DF is associated with the APC gene mutation). Cases of DF of jawbones in association with other conditions like familial adenomatous polyposis, Marfan syndrome, or tuberous sclerosis were also excluded. PDF files of the original articles of either single case reports or multiple cases were obtained and studied for all cases included. Since 1961, a total of 195 records of DF of jawbones were found. 8 9 10 11 12 13 14 15 16 17 18 19 20 21 22 23 24 25 26 27 28 29 30 31 32 33 34 35 36 37 38 39 40 41 42 43 44 45 46 47 48 49 50 51 52 53 54 55 56 57 58 59 60 61 62 63 64 65 66 67 68 69 70 71 72 73 74 75 76 77 78 79 80 81 82 83 84 85 86 87 88 89 90 91 92 93 94 95 96 97 98 99 100 101 102 103 104 105 106 107 108 Details of every case included were methodically reviewed and fed to *Excel* spreadsheet templates. All reviews were done by a single person (the sole author).

For every case, the following features were analyzed: demographic data (number of cases reported annually, age and gender, country, and racial origin), jawbone involved (mandible or maxilla), clinical presentation, treatment, and follow-up course. Simple descriptive statistics were made using the same *Excel* software. Percentages were calculated and the z-test was used to compare percentage scores to see if the difference between them is statistically significant. Due to the simple descriptive nature of data, *p-* values rather than confidence intervals were used to express significance. When data were missing, no statistical methods were used to compensate for them. No sensitivity studies were done to evaluate reliability of the results.

## Results

### Demographic Features

#### Number of Reported Cases Per Year

**Fig. 2:**
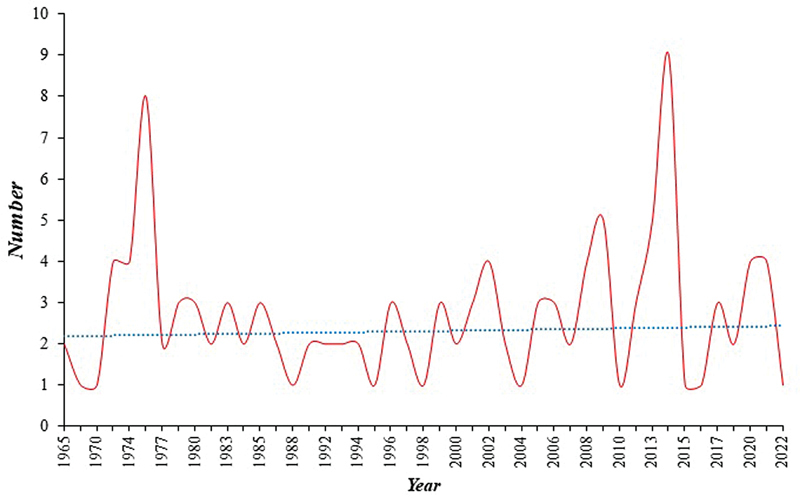
Number of cases of desmoplastic fibroma of jawbones reported per year (dotted line refers to trend line).

Since 1961, a total of 195 cases of DF of the jawbones were reported ( Fig. 2 ), the trend was almost annually continual. After excluding the years when large case series were retrospectively reviewed (namely 1968, 1976, 1981, 1986, 1989, 1990, 2012, and 2019), it was found that the average number of cases that were reported every year amounted to 2.7 cases per year.

#### Age and Gender

**Fig. 3:**
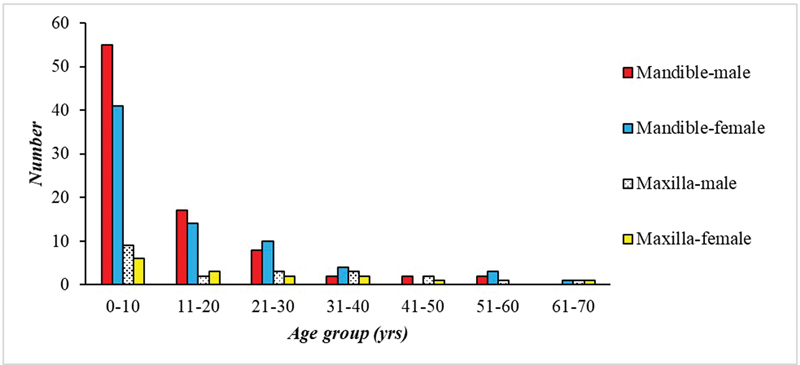
Age and gender distribution of cases of desmoplastic fibroma of the jawbones ( *n* = 195).

Of the 195 reviewed patients, there were 107 (54.9%) males (86 mandibular and 21 maxillary) and 88 females (73 mandibular and 15 maxillary) ( Fig. 3 ), giving an overall female:male ratio of 1:1.3 (1:2 mandibular and 1:4 maxillary). The average age at the time of initial diagnosis, for DF of both jaws, was 13.5 years (standard deviation [SD]: 14.4). It ranged from 2 months to 68 years, with a median value of 8 years. The average age for mandibular and maxillary cases was 12.1 (± 13.2 SD) and 20.5 (± 13.0 SD), respectively. The peak frequency for the whole sample (111; 56.9%) was in the first decade ( *p* < 0.05; Fig. 3 ), this was also the case for both of the mandibular and maxillary cases (96; 60.4% ( *p* < 0.05) and 15; 41.7%, respectively).

#### Country and Racial Origin

**Fig. 4:**
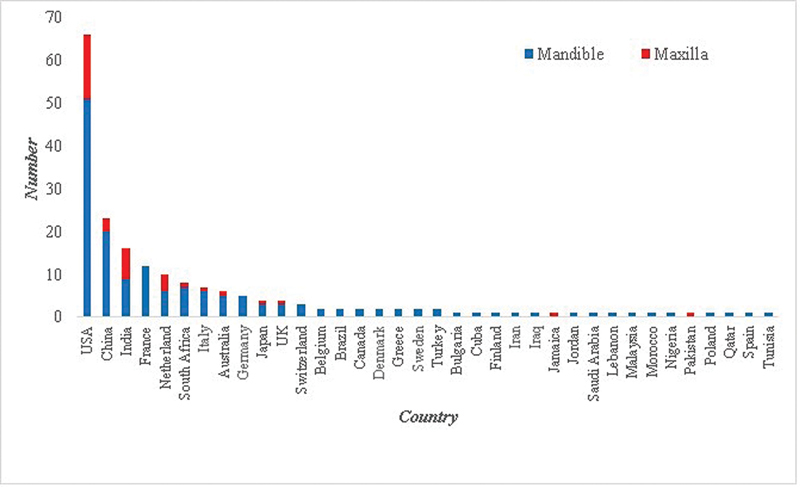
The document country of origin for cases of desmoplastic fibroma of jawbones ( *n* = 195 cases).

**Fig. 5:**
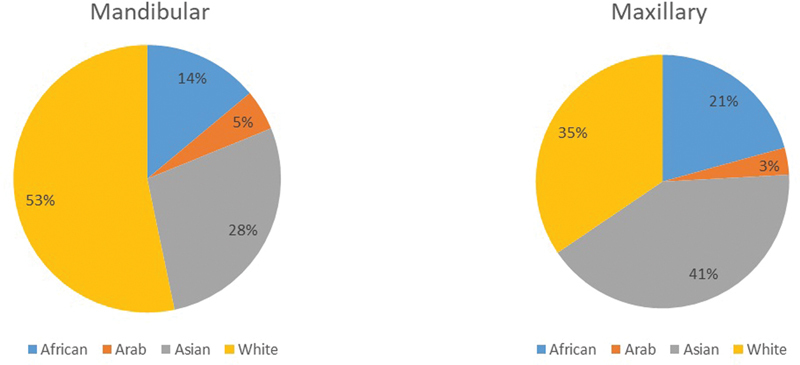
Racial distribution for cases of desmoplastic fibroma of jawbones ( *n* = 151 cases).

The country of origin for the 195 cases, in descending order, is shown in Fig. 4 . For countries known to have citizens from several racial origins (Australia, Canada, South Africa, United States, and United Kingdom) with 86 cases, the racial origin could be obtained for 42 out of 86 cases. The net number of cases, affecting both jaws, with documented racial origin was 151 cases, distributed as follows in descending order: 46 (30.4%) white, 41 (27.1%) Asians, 23 (15.2%) African, and 7 (4.6%) Arab. For individual jawbones, the racial distribution ( Fig. 5 ) had a similar pattern for mandibular cases. However, maxillary cases showed a higher percentage (12; 41%; *p* < 0.05) of Asian racial origin ( Fig. 5 ).

### Jawbone Distribution of Lesions

Of the 195 cases of DF cases, the most commonly affected jaw was the mandible in 159 (81.5%) cases. Maxillary bone was affected in 36 (18.5%) cases ( *p* < 0.01). Of the reported 159 mandibular cases, 125 (78.6%) had information about the exact anatomical subsite. The posterior aspect of the mandible was found to be affected in 114 (91.2%) cases ( *p* < 0.05), and the remaining 11 (9.8%) mandibular cases showed involvement of both anterior and posterior aspects of the mandible; there were no isolated anterior mandibular sites. The most commonly involved mandibular subsite was a combination of “ramus plus angle plus body” (25; 20%; *p* < 0.05) followed by “isolated body” subsite (21%), followed by other subsites ( Table 1 ).

**Table 1:** Jawbone distribution of desmoid fibroma in cases with information about the exact anatomical subsite ( *n* = 157 cases)

Mandibular	No. (%)	Maxillary	No. (%)
**Ramus + angle + body**	25 (20)	Anterior (sinonasal)	5 (15.6)
**Body**	21 (16.8)	Alveolar ridge—posterior	4 (12.5)
**Angle + body**	17 (13.6)	Whole (alveolar ridge + body and sinus)	4 (12.5)
**Ramus + angle**	10 (8)	Palate–posterior	3 (9.4)
**Condyle neck + ramus + angle + body**	9 (7.2)	Whole (alveolar ridge + body and sinus + orbital floor)	3 (9.4)
**Ramus**	8 (6.4)	Posterior (alveolar ridge + body and sinus + orbital floor + infratemporal fossa)	2 (6.3)
**Parasymphysis**	7 (5.6)	Palate–anterior	1 (3.1)
**Body + parasymphysis**	5 (4)	Whole (alveolar ridge + body and sinus + nasal + orbital floor + intracranial)	1 (3.1)
**Hemi-mandible**	5 (4)	Anterior (body and sinus)	1 (3.1)
**Angle**	4 (3.2)	Anterior (body and sinus + orbital floor)	1 (3.1)
**Angle + body + parasymphysis + symphysis**	4 (3.2)	Posterior (alveolar ridge + body and sinus + hard palate + soft palate)	1 (3.1)
**Condylar neck + ramus**	3 (2.4)	Posterior (alveolar ridge-molar area)	1 (3.1)
**Angle + body + parasymphysis**	2 (1.6)	Posterior (alveolar ridge-molar + premolar)	1 (3.1)
**Ramus + angle + body + parasymphysis + symphysis**	2 (1.6)	Posterior (body and sinus + pterygoid plates)	1 (3.1)
**Body + parasymphysis + symphysis**	1 (0.8)	Posterior (body and sinus + zygoma)	1 (3.1)
**Condylar head + condylar neck + ramus**	1 (0.8)	Whole (alveolar ridge + body and sinus + nasal + orbital floor + nasopharynx + intracranial)	1 (3.1)
**Condylar head + condylar neck + ramus + angle**	1 (0.8)	Whole (alveolar ridge-molar + anteriors)	1 (3.1)
**Total**	**125 (100)**		**32 (100)**

Of the 36 maxillary cases, 32 (88.9%) had information about the exact anatomical subsite. The anterior aspect of the maxilla was mostly affected in 17 (53.1%; *p* < 0.05) cases, followed by 8 (25%) cases affecting both of anterior and posterior aspects of the maxilla. Anterior sinonasal maxillary subsite was in five (15.5%) cases, followed by the posterior alveolar ridge in four (12.5%; *p* < 0.01) ( Table 1 ).

The side of the gnathic skeleton affected by DF was documented in 134 (68.7%) cases, of these 108 (67.9%) were mandibular and 26 (72%) were maxillary. Mandibular cases were more common on the left side (61; 56%, *p* < 0.05), whereas maxillary cases were slightly more common on the right side (14; 53.8%, *p* < 0.05).

### Clinical Presentation

Among the 195 cases included, pain or tenderness was reported 28 (14.4%) times in 22 (13.8%) mandibular and 6 (16.7%) maxillary cases. Trismus was reported in 19 (10.7%) mandibular cases and 1 (2.7%) maxillary case (where there was involvement of the pterygoid area). Ulceration of the intraoral part of the reported DF cases was reported in four (2.5%) mandibular and two (5.6) maxillary cases. None of the reviewed 195 cases showed any form of facial skin ulceration or discoloration.

Information about the individual lesion size was available in 77 (39.5%) reports. It ranged from 8 mm to 10 cm, in greatest dimension, with an average of 4.4 cm (± SD 2.3 cm) and a median of 4 cm. The size of mandibular lesions ranged from 8 mm to 10 cm in greatest dimension, with an average of 4.4 cm (± SD 2.4 cm), and a median of 4 cm. On the other hand, the size of maxillary lesions ranged from 1.9 to 9 cm in greatest dimension, with an average of 4.3 cm (± SD 1.8 cm), and a median of 4 cm. The duration of onset for all DFs varied from 1 week to 60 months with a median duration of 3 months. There was a weakly positive correlation (correlation coefficient of 0.2) between the duration and the size of DF for mandibular and maxillary bones.

The lesions' consistency was mentioned in 70 (35.9%) DF cases, (56 (35.2%) mandibular and 14 (38.9%) maxillary). The most frequently reported consistency was “firm” in 48 (68.6%; *p* < 0.05) cases, 38 (67.9%; *p* < 0.05) mandibular, and 10 (71.4%) maxillary. This was followed by “hard” in 20 (28.6%) cases, 17 (30.4%) mandibular, and 3 (21.4%) maxillary. Only one (1.4%) case was reported as soft and belonged to the maxillary group of lesions.

### Radiographic Appearance

Information about the radiographic and/or other imaging modalities (CT, MRI, or US) was available for 131 (67.2%) cases (114 (71.7) mandibular and 17 (47.2%) maxillary cases). All cases showed variable degrees of nonspecific bone destruction of the involved region. In 11 (9.6%; *p* < 0.1) cases of mandibular DF, bone formation at the lesions' periphery giving a “sunray appearance” was reported; this was not reported in any maxillary case.

### Provisional Diagnoses

A provisional clinical diagnosis preceding the incisional biopsy was mentioned in only 18 (9.2%) reports. It included cyst (4 cases), central giant cell granuloma (4 cases), sarcoma (4 cases), odontogenic tumor (2 cases), tuberculosis (2 cases), and salivary tumor (1 case).

### Treatment and Outcome

Information about treatment was available in 193 (98.9%) cases (157 mandibular and 36 maxillary). Of these, 162 (83.9%) received a single treatment modality (135 (85.9%) mandibular and 27 (75%) maxillary cases), and 27 (14.5%) received more than one treatment modality (19 (12.1%) mandibular and 9 (25%) maxillary). Two mandibular cases, once initially diagnosed as DF by incisional biopsies, were kept under observation without any active intervention, and one patient with mandibular DF refused treatment.

For mandibular cases, single-modality treatments used were as follows, in a descending order: surgical (129 cases), neoadjuvant cytotoxic chemotherapy (5 cases), and neoadjuvant radiotherapy (1 case) ( Table 2 ). The combined cure rate (with no recurrence at the time of individual case reporting) for all treatment modalities was 74.8% ( *p* < 0.05). The most effective treatment was surgical (97; 75.2% ( *p* < 0.05) disease-free rate after a mean follow-up period of 102 months). Composite resection was the most successful (21; 95.5% ( *p* < 0.01) disease-free rate after a mean follow-up period of 36 months; Table 2 ). De-bulking, enucleation, and curettage were the least effective methods with recurrence rates of 100, 57.1, and 42.9%, respectively. Spontaneous resolution was reported in one case after a follow-up period of 36 months. Mandibular cases that received a multimodal treatment were 19 cases ( Table 3 ); our results showed that neoadjuvant cytotoxic chemotherapy followed by surgery (4 of 5 disease-free) and neoadjuvant cytotoxic chemotherapy followed by surgery followed by adjuvant cytotoxic chemotherapy (3 of 4 disease-free) were the most effective combinations for mandibular DF ( *p* < 0.05).

**Table 2:** Treatment modalities for DF of jawbones with information about the exact treatment ( *n* = 193 cases)

	Treatment			No.	Disease-free (%)	Follow-up (mo)	Recurred	Follow-up (mo)
**Mandibular**	Surgery	Enucleation	7	3 (42.9)	96	4 (57.1)	60
		Curettage		14	8 (57.1)	32.6	6 (42.9)	15.7
		Debulking		3			3 (100)	41
		Excision-unspecified		14	7 (50)	84	7 (50)	24
		Resection	Unspecified	29	25 (86.2)	72	4 (13.8)	30
			Upper marginal	9	8 (88.9)	34	1 (11.1)	36
			Lower border	1	1 (100)	54		
			Partial	30	24 (80)	24	6 (20)	24
			Composite	22	21 (95.5)	36	1 (4.5)	18
	Total surgical			129	97 (75.2)	Mean = 54 ± 26.9	32 (24.8)	Mean = 31 ± 14.5
	Neoadjuvant cytotoxic chemotherapy			5	3 (60)-stable	31	2 (40)-progressive	21
	Neoadjuvant radiotherapy			1	1 (100)	30		
	Total single modality			135	101 (74.8)	Mean = 49.4 ± 25.7	34 (25.2)	Mean = 29.9 ± 13.9
	None (observation and refused)			3			3 (100)	17
	Multimodality			19	10 (52.6)		9 (47.4)	
	*Total*			*157*	*111 (70.7)*		*46 (29.3)*	
**Maxillary**	Surgery	Enucleation		1	1 (100)	??		
		Curettage		3	2 (66.7)	36	1 (33.3)	36
		Excision-unspecified		5	4 (80)	69	1 (20)	36
		Resection	Unspecified	5	5 (100)	52.5		
			Partial	10	8 (80)	44.2	2 (20)	40
			Complete	1	1 (100)	24		
	Total surgical			25	21 (84)	Mean = 45.1 ± 16.9	4 (16)	Mean = 37.3 ± 2.3
	Neoadjuvant cytotoxic chemotherapy			2	1 (50)	76	1 (50)	9
	Total single modality			27	22 (81.5)	Mean = 50.3 ± 19.7	5 (18.5)	Mean = 0.3 ± 14.3
	Multimodality			9	3 (33.3)-FOD	54	3 (33.3)-progressive	10
					3 (33.3)-stable	10		
	*Total*			*36*	*28 (77.8)*	*Mean = 39.3 ± 23.6*	*8 (22.2)*	*Mean = 26.2 ± 15.3*

Abbreviation: DF, desmoplastic fibroma.

**Table 3:** Multimodal Treatments for DF of jawbones for cases with information about the exact treatment ( *n* = 27 cases)

	Multimodalities		Outcome					
		No.	D-F	Fu (mo)	Regression	Fu (mo)	Progression	Fu (mo)
**Mandible**	*Neoadjuvant chemotherapy-surgery—adjuvant chemotherapy*	4	3	51			1	13
	*Surgery—adjuvant hormonal therapy*	3			3	30		
	*Surgery—adjuvant chemotherapy*	4	2		2	12		
	*Surgery—adjuvant chemotherapy + adjuvant hormonal*	1			1	31		
	*Neoadjuvant chemotherapy—surgery*	5	4	67	1	29		
	*Surgery—adjuvant radiotherapy*	2	1	30			1	30
	**Total**	**19**	**10**	**Mean = 148**	**7**	**Mean = 102**	**2**	**Mean = 43**
**Maxilla**	*Surgery—adjuvant chemotherapy*	3			1	6	2	
	*Surgery—adjuvant radiotherapy*	4	3				1	12
	*Neoadjuvant chemotherapy—adjuvant radiotherapy*	1			1	9		
	**Total**	**8**	**3**		**2**	**Mean = 15**	**3**	**Mean = 12**

Abbreviations: DF, desmoplastic fibroma; D-F, disease-free; Fu, follow-up.

For maxillary cases, single-modality treatments used were surgery (25 cases) and neoadjuvant cytotoxic chemotherapy (2 cases). The combined cure rate (with no recurrence at the time of individual case reporting) for all treatment modalities was 28 (77.8%, *p* < 0.05) cases ( Table 2 ). The most effective treatment method was surgical treatment (21; 84% disease-free rate after a mean follow-up period of 45 months; *p* < 0.05), with partial and complete maxillectomies being the most successful (80 and 100%, respectively) with disease-free rates of 44.2% after mean follow-up periods and 24 months ( Table 2 ). Maxillary cases that received multimodal treatment were eight cases ( Table 3 ). Our results showed that surgery followed by adjuvant radiotherapy was the most effective combination (three out four disease-free) for maxillary cases.

## Discussion

DF is a benign, rare, spindle-cell neoplasm that can affect the human bone most frequently the jaw, skull, femur, and pelvis. This tumor has a destructive local growth and a predilection for recurrence after inadequate therapy, but without the ability for metastases. 109 The histopathological features of DF include proliferation of marginally atypical, innocuous-appearing, slender spindle cells, whose nuclei are round or fusiform in shape, and their chromatin is finely divided, with very few, if any, mitoses. The cytoplasm is eosinophilic, linear, ill defined, and may be wavy. The stroma shows abundant independent dense fibers and bundles of collagen, with cells and collagen fibers being oriented in either a linear or a twirling arrangement. The nuclear overexpression of β-catenin is a useful diagnostic tool. 110 At a genetic level, DF comprises at least two different clinico-pathological entities: sporadic DF and DF associated with germline mutation of *APC* . 111 DFs are driven by changes of the *Wnt/APC/β* -catenin pathway, sporadic variants are associated with somatic mutations of *CTNNB1* , and germline mutations of *APC* and somatic mutations of *CTNNB1* are probably mutually exclusive.

DF is a distinctly rare disease; it has an estimated incidence of 5 to 6 cases per 1 million of the population per annum, 3 of which the majority arise from visceral or abdominal wall soft tissue; head and neck lesions comprise 15% of cases. 112 DF comprises less than 1% of all bone tumors including benign bone neoplasms. 113 To the best of our knowledge, the incidence of DF of jawbones has not yet been published. In our review of cases published during a period of more than 60 years, we found that two to three new cases are published every year, which further confirms the rarity of this entity.

We found that DF of jawbones shows a slight male gender predilection; this was true for individual mandibular or maxillary lesions, and for the combined lesions affecting both jaws. This is in general agreement with previous series of DF involving either the whole body 114 or jawbones. 68 115 116 However, it disagrees with other studies of DF of jawbones that found an equal gender distribution. 78 The difference between our findings and those of others could be attributed to the fact that, unlike our study, previous reports concentrated on the mandibular jawbone or on the pediatric group of age.

The peak frequency of age, in our study, for DF of individual, and combined, mandibular or maxillary jawbones and the whole sample was in the first decade. The average age for DF of both jaws was 13.5 years, and for mandibular and maxillary cases was 12.1 and 20.5, respectively. This differs from one previous study, 115 but is similar to others. 68 80 DFs of the whole body 116 and of the head and neck 103 are traditionally described as being more common in patients under 30 years of age.

We found that pain, trismus, and intraoral ulceration were infrequent features of jawbone DF, this is in general agreement with others. 68 71 116 117 In our review, DF of both jawbones together, and for the mandible alone, was more frequent among white people. However, maxillary DF was more frequent among Asians. One review 71 has shown DF to be more prevalent among whites. However, to the best of our knowledge, racial distribution of maxillary DF has not been investigated earlier.

All reviewed cases showed variable degrees of nonspecific bone destruction of the involved region. When an extensive tumor affects the jawbones, its clinical and radiological appearance may cause diagnostic difficulties. Bony destruction accompanied by sizable facial or upper cervical swelling with intact skin and oral mucosae would suggest the presence of an aggressive intraosseous lesion. Therefore, DF could be misdiagnosed as other more common jaw lesions that exhibit rapid and aggressive clinical and radiographic behavior notably sarcomas and giant cell tumor. 85 106 109 110 For the best management of DF, a clear diagnosis by histologic and clinical examination is mandatory; therefore, adequate biopsy is binding before deciding on the treatment plan. Our review showed that several treatment modalities have been tried for jawbone DF singly or in sequential combinations. These methods included surgery (of variable extents), neoadjuvant and/or adjuvant cytotoxic chemotherapy, neoadjuvant and/or adjuvant cytotoxic radiotherapy, and adjuvant hormone therapy.

DFs are relatively sizable tumors, and we found an average of 4 cm in greatest diameter of both maxillary and mandibular lesions. Although one previous series found no correlation between the duration of the symptoms and the extension of the tumor mass, 68 we found a weakly positive correlation (correlation coefficient of 0.02) between the duration and size of DF of the two jaws, singly and combined. The size of a tumorous growth will naturally dictate the extent of surgical ablation and the need of other treatment modalities. Therefore, the majority of our cases were treated with relatively aggressive composite resections with or without adjuvant or neoadjuvant chemotherapy or radiotherapy (see below).

DF is notorious for being a recurrent disease, some investigators have suggested that the high recurrence of DF is not only a result of inadequate surgical excision but also related to the “innate biology” of the lesion. 47 They have suggested that DFs exhibiting high cellularity have higher recurrence rates than those with lower cellularity. For mandibular and maxillary DFs, the combined cure rate for all treatment modalities was 74.8 and 81.5%, respectively, with surgery, notably composite resection, being the most effective treatment method. The extent of surgical ablation of bony DF has been a source of disagreement. While some operators prefer minimally invasive procedures or limited local excision, others recommend composite wide-margin resection. It has been reported that wide resection showed minimal or no recurrence, compared with minimal surgery or limited local excision. 61 71 We have also found that these minimally invasive procedures are the least effective methods with considerably high recurrence rates.

Although extensive and mutilating composite resection of a benign neoplasm is not an easy decision, when necessary it has to be done as DF can result in a lethal outcome. 118 Therefore, complete removal of DF with a safety margin of histologically tumor-free tissue (R0 resection) is usually recommended. 118 When a jawbone is involved, and according to the tumor extension, surgery may be limited to removal of the periosteum or may involve resection of parts of the bony cortex. 68 Lesions that are more extensive may require partial or complete resection.

We have found that the vast majority of DFs affect children; this age distribution renders treatment planning and decision making a difficult task. During facial growth, the maxilla and mandible play crucial roles. Avoidance of growth disturbance of the face due to extensive jawbone ablation is a critical issue concerning surgery of DF in children. Although immediate free-flap reconstruction can reduce the destructive side effects of the tumor eradication, all possible deleterious effects of treatment need to be considered. For these reasons, extensive tumors require a multimodal, multidisciplinary treatment approach. 49 The therapeutic alternatives that can be used for DF include chemotherapy (e.g., vincristine/actinomycin C/cyclophosphamide or methotrexate/vinblastine), 119 nonsteroidal anti-inflammatory drug therapy and therapy with hormonal agents (antiestrogen), 120 selective tyrosine kinase inhibitors (e.g., imatinib mesylate), and interferon-α. 121

Radiotherapy is another therapeutic modality for DF. Adjuvant radiotherapy is indicated in the treatment of patients with positive margins following wide excision of recurrent disease. The role of adjuvant radiotherapy in patients with positive margins following resection of primary disease is controversial, and should be based on a balanced evaluation of the potential morbidity from radiotherapy compared with the potential morbidity of another local recurrence. 122 Little has been published about the outcome of radiotherapy. It has been shown that radiotherapy, added to surgery with residual disease or to surgery for recurrent disease, led to significant improvement in local control of DF. 123 In the current review only a very limited number of cases of DF of jawbones were treated with radiotherapy, which further precludes obtaining meaningful conclusions regarding its efficacy.

Recently, the approach to treating musculo-aponeurotic DF has moved significantly from surgery to a “wait-and-see” strategy for nonevolving disease. 124 The European Pediatric Soft tissue sarcoma Study Group recommends first adopting a wait-and-see strategy to assess the tumor's rate of growth (or potential spontaneous regression), especially for tumors not at life-threatening sites. Only in cases of frank tumor progression, increasing pain/symptoms, or that involve life-threatening sites, treatment should be considered. 125 The proposed first treatment in such cases is low-dose intravenous methotrexate plus vinblastine for at least 6 months. The role of surgery after systemic therapy remains an open question. As for DF of the jawbones, this recent approach is not yet reflected in the recent literature since we have found only one case of spontaneous resolution after a follow-up period of 36 months.

Weaknesses of this study include that fact that some of the features analyzed were not mentioned in some of reported cases. However, this was accounted for and analyses were made on available data after omission of missing parameters. The other weakness stems from the fact that cases published in non-English language were not included.

In summary, DF is a rare benign locally aggressive bone tumor with a high rate of recurrence. We have reported a case of DF of mandibular bone that warranted a comprehensive literature review of this lesion. To the best of our knowledge, this review is the first to delineate difference between mandibular and maxillary DFs. Based on our findings, the recommended treatment consists of complete resection with safety margin followed by adjuvant chemotherapy in mandibular cases, or adjuvant radiotherapy for maxillary cases.
